# Co-regulatory network analysis of the main secondary metabolite (SM) biosynthesis in *Crocus sativus* L.

**DOI:** 10.1038/s41598-024-65870-z

**Published:** 2024-07-09

**Authors:** Mahsa Eshaghi, Sajad Rashidi-Monfared

**Affiliations:** Department of Plant Biotechnology, Faculty of Agriculture, Tarbiat Modares University, Tehran, Iran

**Keywords:** *Crocus sativus*, Co-regulation networks analysis, Hub TFs, Secondary metabolites, Biotechnology, Computational biology and bioinformatics, Gene regulatory networks

## Abstract

Saffron (*Crocus sativus* L.) is being embraced as the most important medicinal plant and the commercial source of saffron spice. Despite the beneficial economic and medicinal properties of saffron, the regulatory mechanism of the correlation of TFs and genes related to the biosynthesis of the apocarotenoids pathway is less obvious. Realizing these regulatory hierarchies of gene expression networks related to secondary metabolites production events is the main challenge owing to the complex and extensive interactions between the genetic behaviors. Recently, high throughput expression data have been highly feasible for constructing co-regulation networks to reveal the regulated processes and identifying novel candidate hub genes in response to complex processes of the biosynthesis of secondary metabolites. Herein, we performed Weighted Gene Co-expression Network Analysis (WGCNA), a systems biology method, to identify 11 regulated modules and hub TFs related to secondary metabolites. Three specialized modules were found in the apocarotenoids pathway. Several hub TFs were identified in notable modules, including MADS, C2H2, ERF, bZIP, HD-ZIP, and zinc finger protein MYB and HB, which were potentially associated with apocarotenoid biosynthesis. Furthermore, the expression levels of six hub TFs and six co-regulated genes of apocarotenoids were validated with RT-qPCR. The results confirmed that hub TFs specially MADS, C2H2, and ERF had a high correlation (*P* < 0.05) and a positive effect on genes under their control in apocarotenoid biosynthesis (CCD2, GLT2, and ADH) among different *C. sativus* ecotypes in which the metabolite contents were assayed. Promoter analysis of the co-expressed genes of the modules involved in apocarotenoids biosynthesis pathway suggested that not only are the genes co-expressed, but also share common regulatory motifs specially related to hub TFs of each module and that they may describe their common regulation. The result can be used to engineer valuable secondary metabolites of *C. sativus* by manipulating the hub regulatory TFs.

## Introduction

Saffron, the dried stigmas of *Crocus sativus* L, belonging to the Iridaceae family is one of the most important medicinal plants which is known as golden condiments in the world. It is also used in textile dye or perfumery ingredients^1^. *C. sativus* is a male-sterile and triploid plant with three homologous sets of chromosomes (2n = 3x = 24) that is cultivated by vegetative corms^2^. It is believed that the original *C. sativus* cultivation most probably developed within the area of Iran, Turkey, and Greece. Nowadays, the largest producer countries for *C. sativus* cultivating and saffron industries contain Iran, Spain, India, Greece, Azerbaijan, Morocco, and Italy^3^. Saffron is widely used in herbal folk medicine for the treatment of various illnesses such as cramps, depression, anxiety, cardiovascular diseases, blood pressure, nervous disorders, cancer, atherosclerosis, hepatic damage, and insulin resistance due to its analgesic, sedative, and antioxidant attributes^4–9^. The biochemical analyses have shown some of the important chemical compounds in saffron include apocarotenoids, flavanol, flavonoids, phenylpropanoids, monoterpenes, anthocyanins, saponins and vitamins (especially riboflavin and thiamine)^10,11^. The leading derivatives of apocarotenoids like crocin and crocetin are the representative egg-yolk, yellow pigment producers, while picrocrocin and safranal contribute to providing the stigmas with spicy flavor and aroma, respectively. These compounds have various beneficial health properties^12^.

The primary precursor of apocarotenoid biosynthesis, i.e., isopentenyl diphosphate (IPP), pro-duce in two distinct pathways: (1) the Mevalonic acid (MVA) pathway (in the cytoplasm)^13,14^ and (2) the non-mevalonic acid or plastidic 2C-methyl erythritol 4-phosphate (MEP) pathway^15,16^. In a series of enzymatic steps, isopentenyl diphosphate (IPP) and dimethylallyl diphosphate (DMAPP) converts to Geranylgeranyl pyrophosphate (GGPP), Afterward, GGPP converts to phytoene. Phytoene synthase (PSY) catalyzes the first committed step of apocarotenoid biosynthesis, therefore, the conversion of GGPP to phytoene. Subsequently, phytoene desaturase (PDS), ζ-carotene isomerase (Z-ISO), ζ-carotene desaturase (ZDS), carotenoid isomerase (CRTISO), lycopene β-cyclase (LCYB) and β-carotene hydrolase (BHY) convert phytoene to zeaxanthin, in multiple sequential steps. Zeaxanthin precursor is cleavaged and produced crocetin dialdehyde and 3-OH-β-cyclocitral, this step is catalyzed by the enzyme carotenoid cleavage dioxygenase2 (CCD2). Finally, crocetin dialdehyde converts to different forms of crocin derivatives (which are composed of different glucosyl and gentiobiosyl es-ters) which are carried out by an aldehyde dehydrogenase (ALDH) and glocosyl transferase enzymes (GLTs/UGTs), whereas 3-OH-β-cyclocitral, on the lesser–known pathway, formed picrocrocin and safranal compounds. The final steps of the above-mentioned apocarotenoid biosynthesis are catalyzed by several glucosyl transferases^17–22^. Ecotype variabilities and different regions can influence the content of secondary metabolites and gene expression in plants^23–27^.

Nowadays, transcriptomic datasets obtained from high-throughput expression data technology could be applied to figure out regulatory networks, which can help to improve the recognition of candidate genes with a defined degree of coordinated expression and the identification of candidate genes for specific processes^28,29^. Weighted Gene Co-expression Network Analysis (WGCNA) is a prevalent method to infer correlation and discovery modules from gene networks^30,31^. This procedure has been effectively applied to recognize the gene modules that are related to secondary metabolic and metabolite fluxes in several medicinal plants^32–36^. Network analysis using WGCNA can reveal new insights into relationships across candidate transcription factors and metabolic profiles^37^. Most of the research on the *C. sativus* plant is related to gene expression profiles and secondary metabolites content analysis^23,38–41^, Even though, the secondary metabolite biosynthesis pathways have been addressed in some studies^21,42,43^. However, the basic mechanisms of the co-regulation and network analysis of the apocarotenoids biosynthesis pathway in *C. sativus* are still limited.

Herein, high-quality transcriptome data sets of *C. sativus* stigmas were retrieved from the NCBI SRA database. We demonstrated that WGCNA analysis could be a powerful method to recognize the co-regulatory mechanism and module detection to reveal SM-associated hub genes in *C. sativus*. Furthermore, the gene expression profiles of hub TFs and related genes in notable apocarotenoid modules were analyzed by RT-qPCR in different ecotypes in order to determine the correlation and regulatory effect among them. Thus, this approach provides new insights into identifying efficient interactions between hub genes and their target in complex transcriptional regulatory networks (TRN) in *C. sativus*.

## Results

### WGCNA analysis of transcriptome data of *C. sativus* stigma

**Figure 1:**
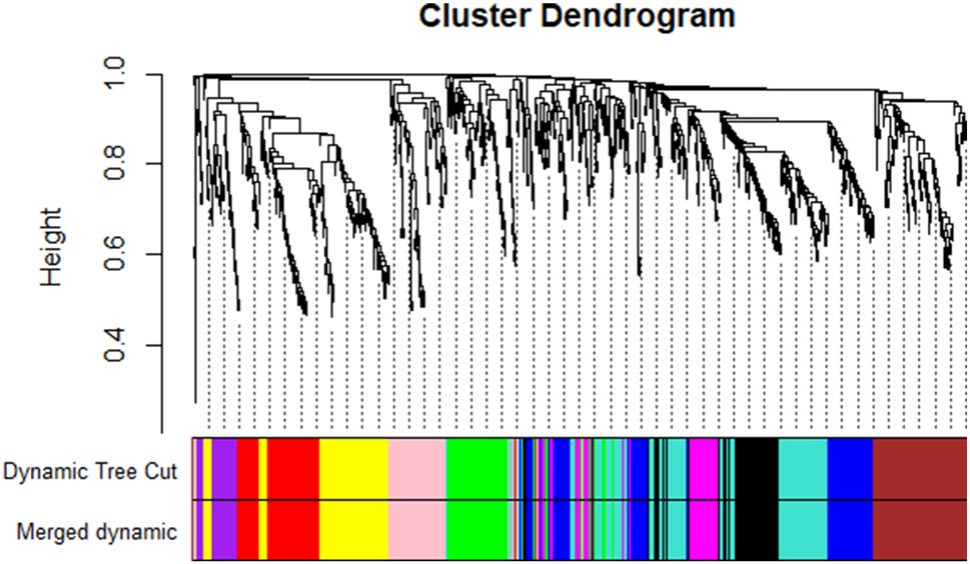
Average linkage hierarchical clustering by the Topological Overlap Matrix and adjacency-based dissimilarity applied to recognize modules. the original modules along with the assigned merged modules are shown with different colors in the below the dendrogram.

The 19 transcriptome data projects (Supplementary Table S1) of the stigma were retrieved from the NCBI SRA database. In total, 435,636,802 RNA-Seq reads of different *C. sativus* stigma samples were processed then 383,360,386 clean reads were obtained after the quality control procedure. To detect the regulatory mechanism of secondary metabolites biosynthesis, gene expression analysis of desired genes involved in main secondary metabolites biosynthesis and several TF gene families in all 19 projects of *C. sativus* were performed then, a co-regulatory network of those genes was constructed. Before carrying out the network analysis, t-SNE analysis was applied to all data sets and assessed patterns and trends of clustering among samples. This analysis explained the local similarities among the data set. t-SNE revealed that the majority of the homologous stigma transcriptomic projects of different ecotypes were grouped into the same category (Supplementary Fig. S1). We used the normalization methods on gene expression data to remove the samples and features with missing information and adjust for sources of noise. We clustered reference batch based on t-SNE analysis. Overall, four batches were found. Removing batches and reducing the unwanted variations improved the identification of outliers and error correction in this study. The results demonstrated that combat-based normalization appeared to give consistent results for building a network. The weighted gene co-expression network analysis (WGCNA) approach is applied for mining specific modules and highly correlated genes related to the secondary metabolites. In order to discover hub genes and co-regulatory networks which play main roles in various secondary metabolites of *C. sativus* stigmas, 19 samples were analyzed by WGCNA. The optimal soft threshold was determined at 5 to construct a scale-free network (Supplementary Fig. S2). The adjacency matrix was computed using the function adjacency, and the topological overlap matrix was established based on dissimilarity between genes by measuring the distTOM = 1-TOM function. Clustered genes were divided into modules, and modules were obtained based on the dynamic cutting tree branches algorithm (Supplementary Fig. S3). Module eigengenes were preform to merge modules whose dissimilarity was below the 0.2 cutoffs (Fig. 1). A total of 11 modules related to the metabolic biosynthesis pathway were gained (Supplementary Fig. S4). The module size ranged from 37 purple to 137 turquoise modules. In order to explore the notable modules that play the regulatory role in the biosynthesis of secondary metabolic pathways in *C. sativus*, KEGG enrichment analysis (www.kegg.jp/kegg/kegg1.html)^44^ was performed in the genes on the interest modules, and considerable enrichment pathways were screened.

The results demonstrated that in the stigma of *C. sativus*: the “Apocarotenoid, Carotenoid biosynthesis, MEP Pathway, Phenylpropanoid biosynthesis, ABC transports” were significantly enriched in brown module. “MVA Pathway, Saponin biosynthesis, monoterpene, Apocarotenoid” were enriched in the blue module. In the turquoise module, the most enrichment pathways were “Flavone and flavanol biosynthesis”. “Flavonoid biosynthesis” and “Apocarotenoid” were enriched in pink and green modules, respectively. Interestingly, numerous genes related to Apocarotenoid biosynthesis were the most significant in brown, blue, and green modules. In addition, the list of the enrichment pathways in the over-representative modules is presented in Table 1.

**Table 1:**
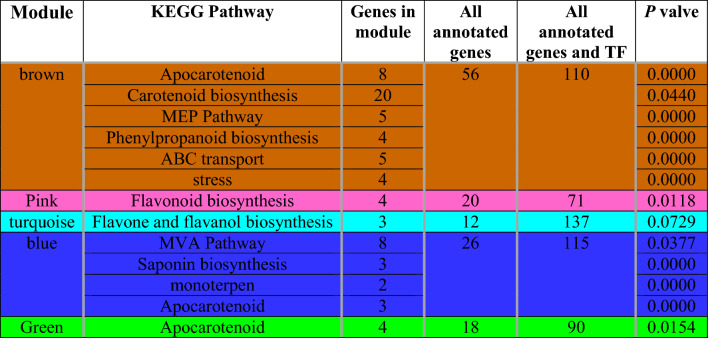
Weighted Gene Co-Expression Network Analysis (WGCNA) for significantly expressed genes in saffron stigma and pathway enrichment analysis of main modules in *Crocus sativus*. The brown, blue and green modules are the specific modules of apocarotenoids. *P* values are computed from Fisher’s exact test (α < 0.05).

### Hub TF recognition and visualization

**Figure 2:**
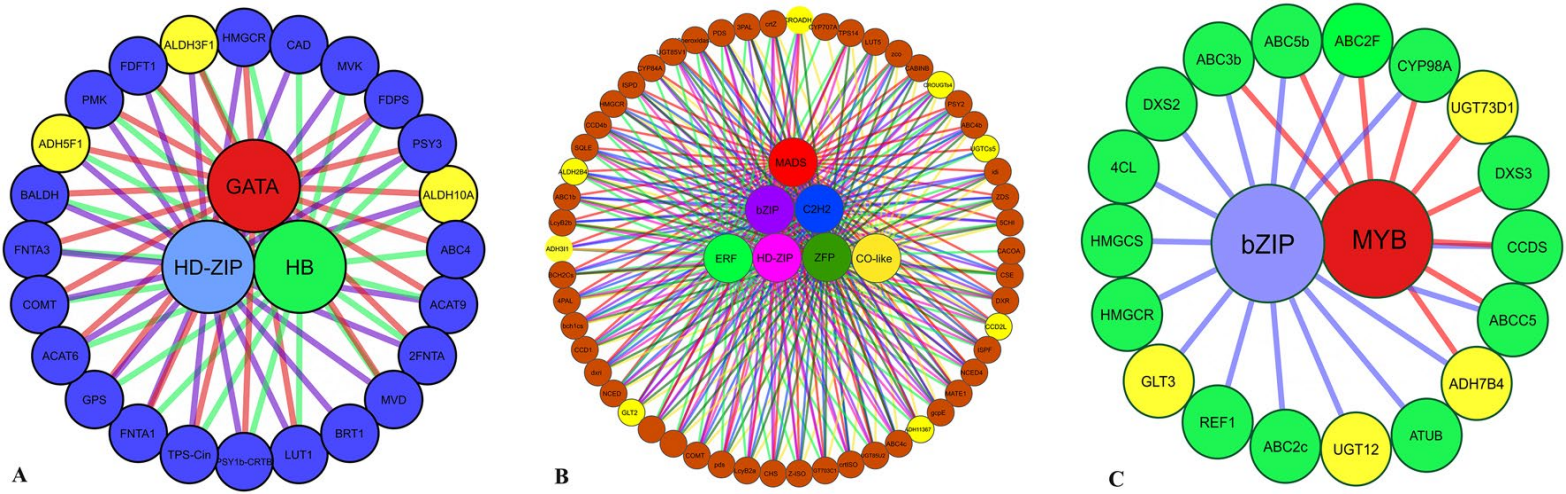
Co-regulated network of hub TFs and structure genes related to secondary metabolites biosynthesis pathway in *C. stativus*. Each TF and the related edge are demonstrated with a specific color. Yellow circles indicate apocarotenoid genes. The notable apocarotenoid modules including blue module (**A**), brown module (**B**), and green module (**C**) are shown, respectively.

In order to identify hub TFs associated with metabolites biosynthesis, hub gene analysis was performed for genes in the modules. Hub gene analysis recognized transcription factors including MADS, C2H2, ERF, bZIP, HD-ZIP, and zinc finger protein in the brown module with the most degree and MCC. bZIP and MYB in the green module were also recognized as hub TFs. The blue module contained HD-ZIP, and HB GATA (Fig. 2 and Supplementary Table S2), and the turquoise module included MIKC and HB hub TFs. MYB-related and NF-YB were also highly co-expressed in the pink module. The results of the PPI network indicated the existence of strong relationships and significant connections among most hub genes. The constructed PPI networks based on the hub TFs of three modules related to apocarotenoid biosynthesis indicated ideal and significant connectivity, which emphasized the effectiveness of our approach to organizing functional modules that comprised a set of proteins having similar functions. Hence, these modules might influence the regulation of secondary metabolites biosynthesis. Therefore, warrants further validation for our findings (Supplementary Fig. S5)**.**

### Promoter analysis

Genes with similar regulatory motifs could be co-regulated. One of the best approaches for the description of co-regulated genes is the determination of common cis-acting elements located on their promoter. The commonly shared motifs were derived as promoter analysis by MEME. According to transcription factor binding sites (TFBSs) screening, multiple unique TFBSs of hub TFs in the promoter regions of co-expressed genes in modules related to apocarotenoids were detected (Table 2, Supplementary Table S5). The cis-elements related to TFs such as HB, MYB, WRKY, and MADS were the most common elements in blue, green, and brown modules, respectively that were hub genes related to apocarotenoids modules and illustrate the critical roles of these factors. Mostly, these TFs play a major regulatory role as positive regulators in the metabolite pathways in plants^29,45^.

**Table 2:**
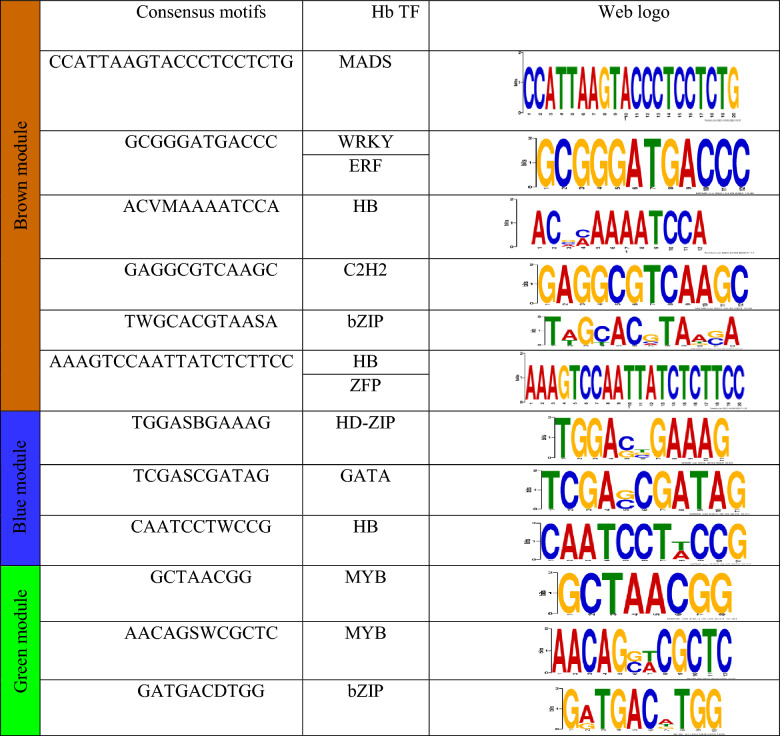
Consensus motifs identified in promoter region of genes of modules involved in apocarotenoids biosynthesis from *C.sativus*.

### Measurement of crocins and picrocrocin content using HPLC

**Figure 3:**
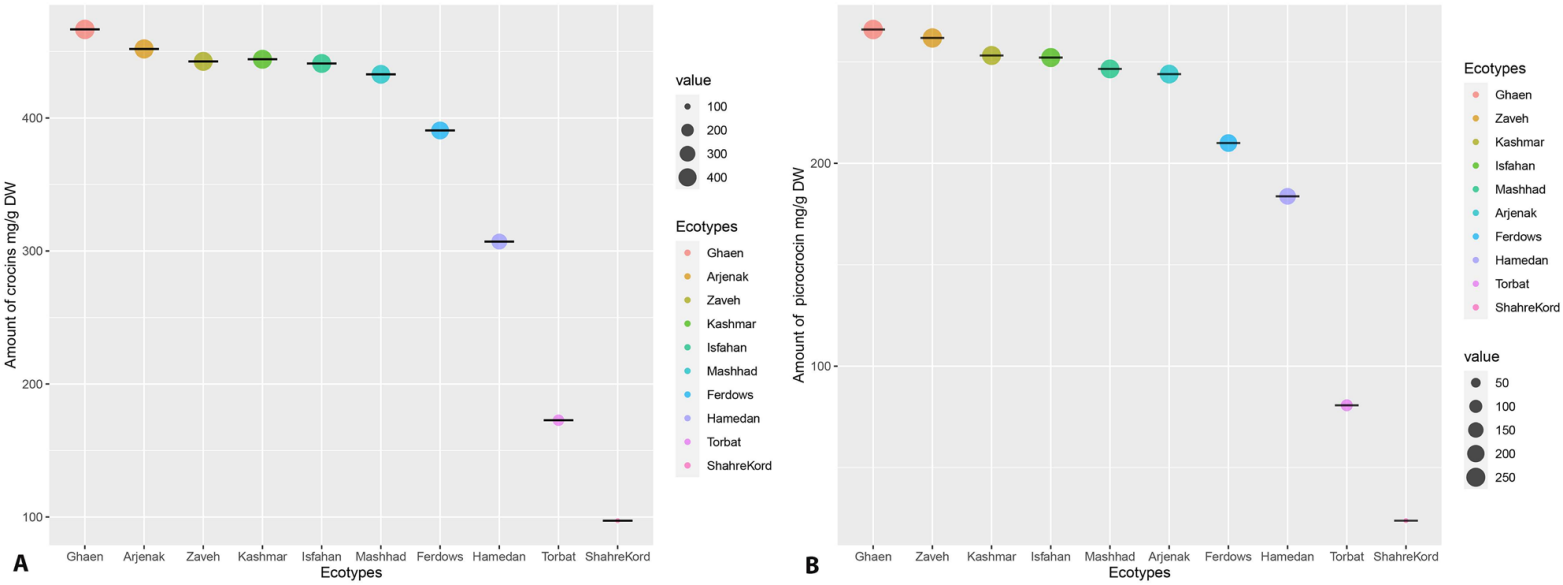
HPLC analysis of the accumulation of crocins and picrocrocin in different ecotypes of C. sativus. The bubble graph shows the contents of crocins (**A**) and picrocrocin (**B**). The size of each bubble indicates the mean of three biological replicates.

The crocins and picrocrocin content of saffron from the studied ecotypes were determined using the HPLC method. According to the HPLC analysis, the concentration of crocins and picrocrocin components differed in the studied ecotypes. The results indicated that the highest concentrations of crocins and picrocrocin as precursors of the saffron aroma components were found in the “Ghaen” ecotype (466.58 and 265.95 mg/g D.W, respectively), whereas the lowest crocins and picrocrocin concentrations of compounds were found in the Shahr-e Kord ecotype (97.20 and 23.84 mg/g D.W, respectively) (Fig. 3). The results illustrated the existence of diversity among ecotypes. Although *C. sativus* has to be reproduced via corms, known as vegetative propagation, it is possible that diversity in the saffron samples is due to the existence of epigenetic variability among ecotypes. The research on the genetic diversity of Iranian *C. sativus* ecotypes from different regions using SSR and SNP markers determined certain genetic variations among *C. sativus* ecotypes and revealed genetic polymorphism among them^46,47^. The results of morphological traits measurement highlighted a phenotypic variation among studied ecotypes. The maximum values of morphological traits, such as fresh stigma weight, dried stigma weight, stigma length, corm weight, horizontal diameter, and flower fresh weight were observed in the Arjenak and Ghaen ecotypes Supplementary Table S3.

### Validation of the relationship between hub TFs and related genes with RT-qPCR

**Figure 4:**
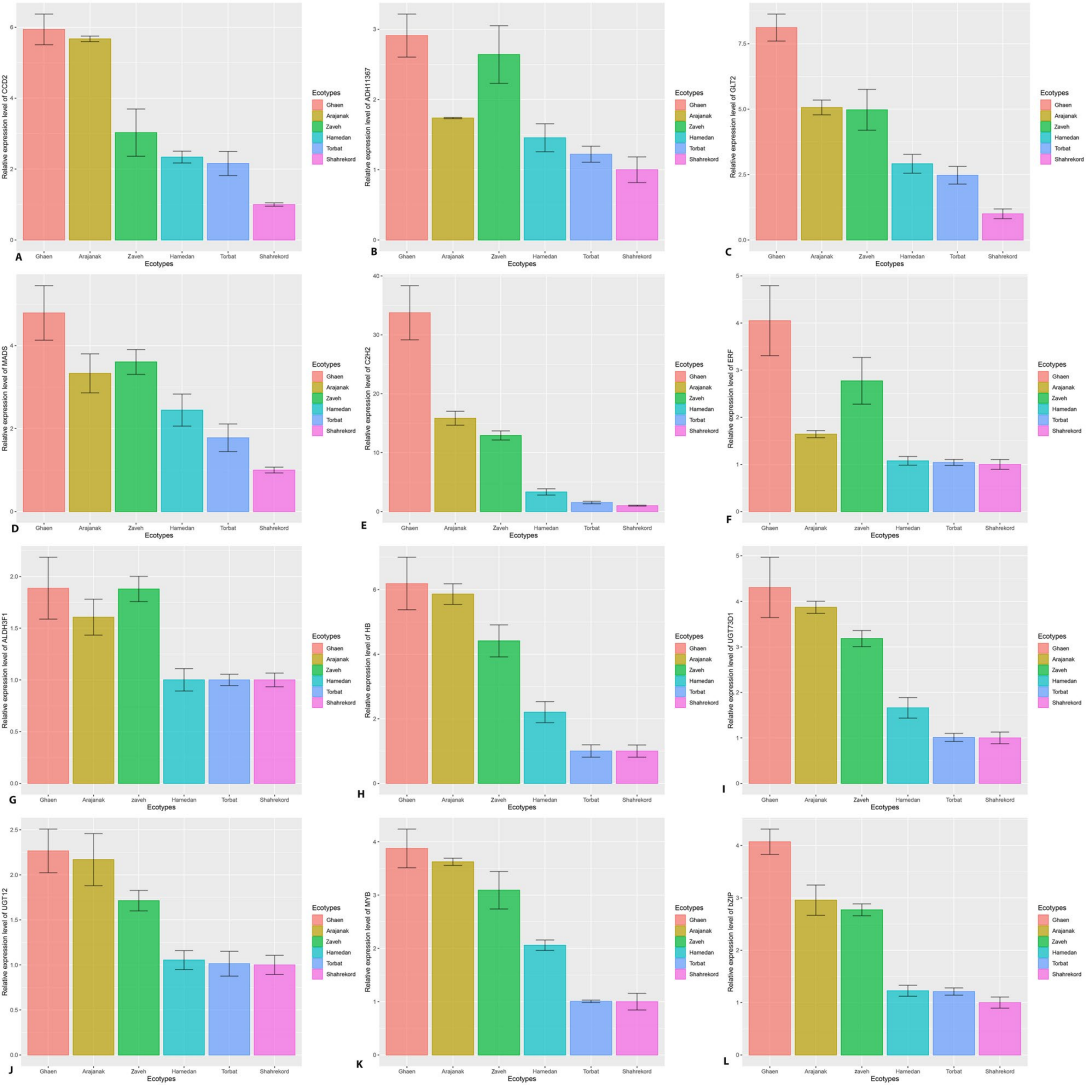
Expression pattern of selected genes from apocarotenoids biosynthetic pathway using RT-qPCR in different high and low metabolite content ecotypes of *C. sativus*. Three hub TFs and three genes in the brown module, namely; CCD2 (carotenoid cleavage dioxygenase 2)(**A**), ADH11367(Aldehyde dehydrogenase11367) (**B**), GLT2(GLT2 Crocetin glucosyltransferase 2) (**C**), MADS(MINICHROMOSOME MAINTENANC AGAMOUS DEFICIENS, Serum response factor)(**D**), C2H2(Cys2-His2)(**E**), ERF(ethylene-responsive factor)(**F**), ADH3F1 (aldehyde dehydrogenase family 3 member F1) (**G**), HB (homeobox)(**H**) and in the blue module., UGT73D1(UDP-glucose-dependent glucosyltransferase-UGT73D1-like protein)(**I**) and UGT12(beta-glucosidase 12) (**J**), MYB (myeloblastosis viral oncogene homolog) (**K**), bZIP (basic-leucine zipper) (**L**) in green, were selected for RT-qPCR analysis. The results indicate the means ± standard error of experiments achieved in triplicate.

**Figure 5:**
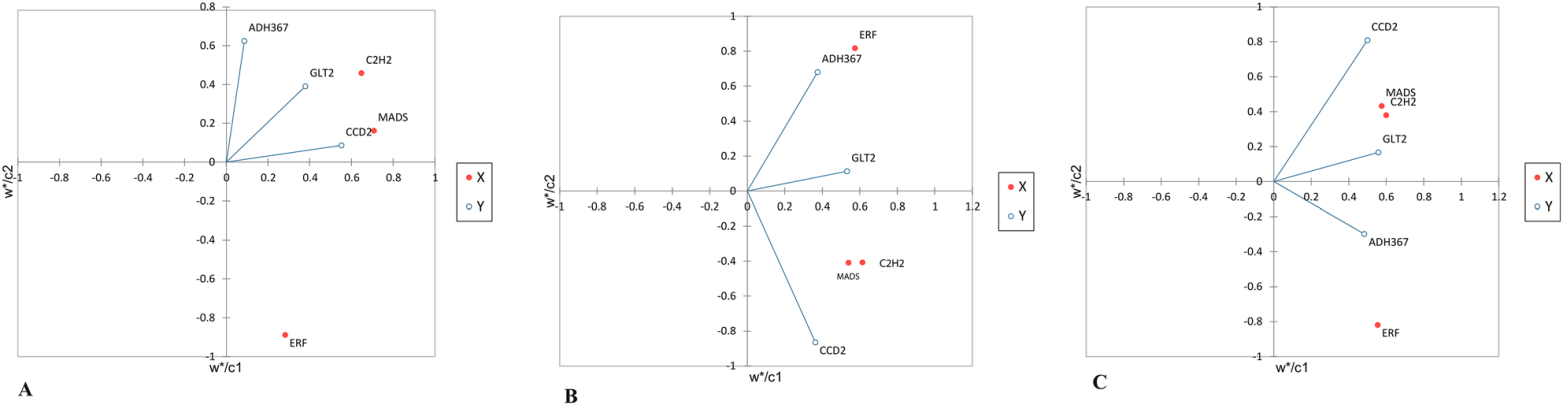
Partial Least Squares regression (PLS) was carried out for determining complex relationships between TFs and dependent genes. The results indicate a strong correlation and more effect between the hub TFs (C2H2 and MADS) and related genes (GLT2 and CCD2) and between ERF and ADH11367. PLS analysis in low- (**A**), high- (**B**), content metabolite and total (**C**) ecotypes.

To verify the chief conclusion drawn from the co-regulation network results for stigma samples, the relative expression levels of hub TFs and candidate genes involved in apocarotenoid synthesis including MADS, C2H2, ERF, CCD2, ADH11367, GLT2 in the brown module, HB and ADH3F1 in the blue module and bZIP, MYB, UGT12 and UGT73D1 in the green module between high-content metabolite ecotypes (“Ghaen”, “Arjenak” and “Zaveh”) and low-content metabolite ecotypes (“Shahr-e Kord”, “Torbat” and “Hamedan”) were determined (Fig. 4). In total, the RT-qPCR results were consistent with the results of the coregulation network analysis. Several research studies have demonstrated a close relationship between metabolite content and mRNA expression level^24,25,48–52^). The RT-qPCR analysis has shown that the relative expression of regulatory TFs and genes tended to give higher up-regulation levels in the high-content ecotypes group than in the low-content ecotypes. As shown in Supplementary Fig. S6, the correlation between hub TFs and genes was positively significant. The relative expression of UGT73D1 which is involved in the synthesis of picrocrocin^53^, in “Ghaen” as a high-content metabolite ecotype was fourfold higher than “Shahr-e Kord” and the correlation between hub TF MYB and UGT73D1 was more than 0.9. The gene expression of GLT2, which is involved in the conversion of crocetin to crocin^19^ increased 8.1 fold in “Ghaen” compared with “Shahr-e Kord” and there was also a significant correlation between hub TFs (MASS, C2H2, ERF) and GLT2 gene (R^2^ > 0.86). Herein, the partial least squares (PLS) regression result illustrated that MADS and C2H2 positively affect related- genes in the apocarotenoid pathway. The PLS result showed that there is also a strong correlation between the C2H2 and GLT2, and ERF and ADH367 (Fig. 5).

It is recommended that the potential participation of a regulatory network exists between hub TFs and related genes in the metabolite biosynthesis pathway among different ecotypes. We also performed PLS analysis to determine the effect of regulon hub genes on their targets in nine transcriptome sequencing data samples from three developmental stages (RED, –2 DAY, and 0 DAY) of stigmas in *C. sativus* which their apocarotenoid contents were also assessed (low and high amounts of apocarotenoids, respectively)^54^. The results showed the strong effect of hub TFs such as MADS, C2H2, ERF, and HD-ZIP on related apocarotenoid genes especially CCD2 and GLT2 (Supplementary Fig. S7). Interestingly, the result of our analysis was nearly consistent with the above results which confirmed the role of the identified hub TFs in the regulation of apocarotenoid biosynthesis in *C. sativus.*

## Discussion

*C. sativus* is a chief source of crucial medicinal metabolites such as apocarotenoids crocin, crocetin, picrocrocin and safranal. Most of the studies in this plant, just have examined metabolite contents assay and gene expression without recognizing relationships among genes and considering regulatory factors including transcription factors. Whereas evaluating the interactions among all regulatory factors can obtain an immense insight into the regulatory mechanisms involved in the secondary metabolite pathways. In this study, we used RNA‑Seq data of *C. sativus* stigma to establish a co-regulatory network for genes involved in the secondary metabolite biosynthesis pathway by using WGCNA analysis. The achievement of integrated regulatory networks can help to understand a more comprehensive knowledge of molecular mechanisms involved in secondary metabolite synthesis. We identified 11 modules related to the metabolite pathways in *C. sativus* and three noticeable modules, namely brown, blue and green modules, which were related to the apocarotenoids synthesis pathway. It is notable that the high connectivity among genes in the same modules can be attributed to the close expression of some genes in the module. In order to discover these potential genes that may be related to the regulation of the biosynthesis of secondary metabolites, different approaches were performed, including assigning modules, functional enrichment analysis of modules, and hub-hub genes recognition. Our findings presented evidence that the regulated hub TF genes may be associated with the coordinated expression of genes involved in the biosynthesis of secondary metabolites. The outcomes also designated that one module can concurrently be related to multiple components, and one component can be regulated by multiple modules.

Hub TFs are considered highly correlated genes for a given module in a biological interaction network. Hub genes were significantly connected in terms of the PPI network. Therefore, TFs with a high degree of connectivity within secondary metabolite modules were also associated at the protein–protein interaction level. In the present study, transcription factors MADS, C2H2, ERF, HB, MYB, and bZIP were recognized as hub genes in modules related to apocarotenoid biosynthesis as showed co-regulated expression with their associated genes in the network. Moreover, the high correlation and positive effect were demonstrated among the genes involved in apocarotenoid production and the hub regulatory TFs in both high- and low-content metabolite ecotypes. PLS analysis showed a considerable effect of C2H2 and MADS on the related genes of apocarotenoid, especially CCD2 and GLT2. Previous research reported that MADS, MYB, MYB-related, WRKY, C2C2-YABBY, and bHLH contain the necessary regulatory machinery for apocarotenoid biosynthesis^40,55^. The TFs including zinc-finger motifs have been previously recognized due to their potential biological functions related to the regulation of apocarotenoid biosynthesis^56^. The screen of MADS, ERF, C2H2, MYB, and zinc-finger protein transcription factors found that these straightly regulate carotenoid genes to definitely and coordinately modulate carotenoid metabolite in plants such as apple fruit, ‘Benin Shogun’, ‘Yanfu 3’ fruit flesh^56–59^. The roles of three of these mentioned hub TFs have been determined and confirmed in previous research in *C. sativus*. For example, the study conducted by Malik et al.^60^ highlighted the role of CstHD, whose expression pattern corresponds to apocarotenoid accumulation in *C. sativus* stigmas. Overexpression of CstHD led to an increase in apocarotenoid content by upregulating biosynthetic pathway genes. Transient expression of CsHD which is identified as hub TFs in our study of co-regulation network, raised apocarotenoid content in metabolism. Furthermore, zinc-finger transcription was identified as a potential regulator of apocarotenoid biosynthesis, with AN20/AN1 being nuclear localized and activating reporter gene transcription in yeast, suggesting its involvement in regulating apocarotenoid biosynthesis^56^. MYB, the main regulatory factor, and genes involved in the apocarotenoid pathway were found in the same modules. Transient overexpression experiments involving MYB genes in *C. sativus* have further confirmed their regulated role in apocarotenoid metabolism. Specifically, these TFs were revealed to regulate apocarotenoid biosynthesis by interacting with the promoters of genes involved in the secondary metabolite pathway^61^. Our findings revealed that more effective and crucial hub TFs such as MADS, C2H2, ERF, HB, and bZIP could be important regulatory genes for regulating the apocarotenoid biosynthesis pathways.

Promoter analysis is a powerful method to describe the presence of common cis-acting elements among genes of the co-expression modules. MYB, WRKY and MADS TFs commonly regulate different genes in the same module which are involved in the metabolite biosynthesis pathway by recruiting to the same cis-acting elements that are placed on their promoters^62–65^. Presence of a few and the same cis-regulatory elements in the promoter region of genes in the same module highlights this point, that genes that participate in the same pathway have a co-regulatory network^66,67^. The results confirmed that “Ghaen”, “Arjenak”, and “Zaveh” not only had the highest expression in RNA transcript level of the hub TFs and related genes but also produced the highest amount of the apocarotenoids among the studied ecotypes. The expression level of the apocarotenoid genes in three ecotypes, “Shahr-e Kord”, “Torbat” and “Hamedan” was notably less than the other ecotypes. A study conducted by Tan et .al 2019 indicated that red and 0 DAY stigmas of the flowering stage, which illustrated the low and high amounts of apocarotenoids, respectively, showed low and high expression of MADS, MYB, zing finger TFs, and UGT2 gene, respectively. A study of the effect of abscisic acid treatment in saffron demonstrated that crocin and safranal contents and the gene expression of CsGT2 and CsLYC were increased coordinately^68^. It seems that a positive correlation would be between metabolite contents and expression profiles in medical plants^24,25,48–52^. A thorough understanding of their mechanism will be the aim of future research. In addition, five genes involved in ABCC Transporters (MATE1, extrusionprotein1b sub-familyCmember4b, Cmember4c, and sub-familyCmember4c) were identified in the brown module. Previous studies were shown ABCC transporters mediated the vacuolar accumulation of crocins in saffron stigmas and were co-expressed with the gene encoding the first dedicated enzyme in the crocin biosynthetic pathway, CsCCD2^69^. This gene had highly coordinated expression and correlation with some of the TFs such as MADS, C2H2, ERF, bZIP, HD-ZIP, and zinc finger protein in the brown module.

Glycosyltransferases play various roles in cellular metabolism by modifying the activities of regulatory metabolites. UGTs catalyze the connections between glucose molecules and specific receptors by glycosidic bonds. Crocetin glucosyltransferase 2 (GLT2) is a glucosyltransferase that plays important role in the biosynthesis of crocins. Herein, GLT2 was more highly expressed in high-content metabolite than in low-content metabolite ecotypes. This gene indicated the highest correlation with associated TFs relative to other genes involved in apocarotenoid biosynthesis based on the test for statistical significance of the correlation coefficient. Several other studies also showed that GLT2 was expressed at high levels in stigmas which facilitate sequential glucosylation of crocetin to crocin in *C. sativus*^19,70,71^. The UGT85 family as stress-regulated UDP-glucosyltransferase-encoding genes have been co-expression with genes related to metabolite biosynthesis in the brown module. The UGT85 family that comprises members related to stress responses and cell cycle regulation in *C. sativus*^72^. In this study, the turquoise module contained the genes of the flavonols pathway that were highly co-expressed. The expression of main flavonols genes including CsGT45 (UGT75P1), and UGT703B1 is highly correlated with the presence of kaempferol^73^. The results illustrated that a close relationship has existed between the expression of CsZCD and stress-regulated genes in the relevant modules based on the increased coordinated expression levels of these genes in stress conditions^74^. So, these findings can predict stress influence on apocarotenoid synthesis by modifying the gene expression and oxidative and peroxidative reactions. Further explorations are clearly required to uncover the relationship between stress conditions and the biosynthesis of apocarotenoids.

## Conclusion

*C. sativus* is the major source of crucial medicinal apocarotenoid metabolites. Herein, we performed a co-regulatory network analysis of the transcriptome projects of *C. sativus*. We identified 11 modules related to *C. sativus* secondary metabolites which three specialized modules covered functions in the apocarotenoid biosynthesis pathway. The result revealed that hub TFs such as MADS, C2H2, ERF, bZIP, MYB, and HB were involved in regulating apocarotenoid biosynthesis. The relative expression of genes involved in apocarotenoid production and associated TFs revealed a strong correlation among various ecotypes, which represented different apocarotenoid contents. Overall, the result of the co-regulatory network analysis of *C. sativus* provided insights into the specific regulatory mechanisms of apocarotenoid biosynthesis at the gene expression level and further provided some candidate hub TFs for regulon engineering.

## Material and methods

### Data collection

Raw transcriptomic sequence data were obtained from the National Center for Biotechnology Information (NCBI) Sequence Read Archive (http://www.ncbi.nlm.nih.gov/sra) database based on the Illumina platform. The accession numbers are included: SRR10028150, SRR10028151, SRR5985561, SRR5985560, SRR5985559, SRR5985558, SRR5985557, SRR5985556, SRR5985555, SRR5985554, SRR5985553, SRR1910567, SRR1909704, SRR1909702, SRR1767302, SRR10028145, SRR10028154, SRR8284572, SRR8284574. The data contained stigma samples related to different ecotypes of *C. sativus*. More details of bio-projects are mentioned in the Supplementary Table S1.

### RNA‑Seq data analysis

Before constructing an integrated regulatory network, data preparation was performed. All processes were run on the Linux operating system (Ubuntu 20.04 LTS). The quality assessment of raw RNA-Seq data was performed by using the FastQC tool (version 0.11.9)^75^. To obtain high-quality reads and remove ambiguous base (N) and adaptor sequences, raw reads were pre-processed by fastp tool (version 0.20.1)^76^.

The clean reads were used for all the downstream analyses. De Bruijn graph-based assembler Trinity package with 32 k-mer was used for de novo assembly^77^. The mapping was performed by using Bowtie 2 (version 2.4.1). To annotate assembled transcripts, we used BLASTx with a cut-off of E-value ≤ 1e − 5 against the NR database, the GO database, and the Swiss-Prot protein database^78^, and to identify transcription factors (TFs) in the *C. sativus* transcriptome data, the PlantTFDB v5.0 database was used^79^. Also, genes and TFs identification along with considering the relevant literature review was performed^40,55,80^. Salmon (version 1.9.0)^81^ was used to quantify the expression of each transcript, and a gene expression matrix was generated based on Transcripts Per Million (TPM) as well as applied the Log2 transformation method to diminution residual variability.

### Batch effects detection and normalization

Batch effects and unwanted variation in RNA-seq experiments are triggered by various factors. Evaluation and removal of batch effects from data could enhance prediction performance in gene expression data. It is essential to normalize the larger merged dataset. We performed tSNE (Rtsne package version 0.16) to recognize the pattern of clustering among different samples. ComBat (SVA package version 3.44.0) function in surrogate variable analysis (SVA) was applied to estimate batch. We used Mean-only adjustment, the popular ComBat model, which adjusts for batch effects and allows the operator to specify the batch name or number to be used as the reference batch^82^.

### Weighted coregulation network analysis and module detection

Regulatory networks and modules of genes were constructed by the WGCNA package in R language^30^. In order to ensure the reliability of the constructed network, the outlier samples were checked and omitted. For this aim, adjacency matrices of expression matrix were considered and sample network connectivity was standardized based on the distances. GoodSamplesGenes function in the WGCNA package was applied to exclude the unqualified genes. The argument maxPOutliers was considered as 0.05 to specify outliers.

To construct scale-free network, a suitable soft-thresholding power was computed based on scale-free topology. The scale-free network is defined with power-law degree distribution and includes several nodes with few interactions and few nodes with high interactions, which are named hub nodes. The approximate scale-free soft threshold was chosen as β = 5. The power 5 exhibited the lowest possible power level at which the scale-free topology fit index curve plateaued, achieving a high value of 0.8, while also attaining a high mean connectivity value. based on this fact, the mean of connectivity falls as power goes up. For module detection, step by step modules function in the WGCNA package was performed with the following main parameters: power = 5, corType = “bicor”, networkType = “Signed Hybrid”, TOMType = “signed”, minModuleSize = 30, deepSplit = 2. The adjacency matrix was converted into a topological overlap matrix (TOM), and the corresponding dissimilarity was measured. The identification of modules was carried out by average linkage hierarchical clustering by the topological overlap matrix of samples. The dynamic tree cut algorithm was used to detect clusters in the hierarchical dendrogram, then module eigengenes were applied to merge highly similar modules.

### Detection of hub genes

The assignment of intramodular connectivity or association between a gene within a specific module and other genes in that module was defined by module membership (kME). MEDissThres was considered as 0.20 and kME threshold was considered > 0.8. Genes with high module membership (kME > 0.8) were good representatives of the overall expression profile in the module and genes with high module membership tend to be “hub” genes in the module (high intramodule connectivity) in our study. To prevent the phenomenon of fuzzy clustering, we considered high KME values. The applied threshold ensures the selection of genes with strong module membership, indicating their importance in the network's structure and function. Also, the genes with the highest association were known as hub genes. CytoHubba plugin of Cytoscape software (version 3.9.1)^83^ was applied to obtain hub genes in each module using Maximal Clique Centrality (MCC) and degree methods for topological analysis. STRING online tool (Version: 12.0) (https://string-db.org/)^84^was used to determine the possible protein interactions network for the hub genes. The background file was chosen monocot genome annotation information to compare the interactions. PPI network analysis recognizes whether co-expressed hub TFs in each module are still significantly connected, according to PPI data.

### Promoter analysis and TFBSs recognition

Homology search between apocarotenoid transcripts and *C.sativus* draft genome (GCA_029339355.1) was performed by BLASTn with the E-value < 1e^−10^ with more than 50% identity then, 1500 bp upstream from TSS was extracted as promoter region of genes involved in apocarotenoid biosynthesis . Motifs recognition of putative consensus motifs was performed using MEME online software (version 5.5.4)^85^ providing letter-probability matrices and statistical modeling methods. Consensus motifs were analyzed by PLACE^86^ and PlantCARE^87^ databases to find putative TFs which relate to these motifs.

### Permission to collect native plant material

This research was based on the PhD research proposal of Mahsa Eshaghi. As the formal project proposal was reviewed by Faculty of Agriculture of Tarbiat Modares University, then received the approvals for conducting the research consistent with local and national regulations (with proposal No. 89852). After that, we obtained permission from the land manager to collect plant corms, then they were planted in the Laboratory of Agriculture and Natural Resources Research and Education Center of Chaharmahal and Bakhtiari Province, Shahrekord, Iran. In the present study, all methods were carried out following relevant guidelines and regulations. Ethical approval or consent was not required for this study because no endangered or protected species were involved.

### Plant cultivation

**Figure 6:**
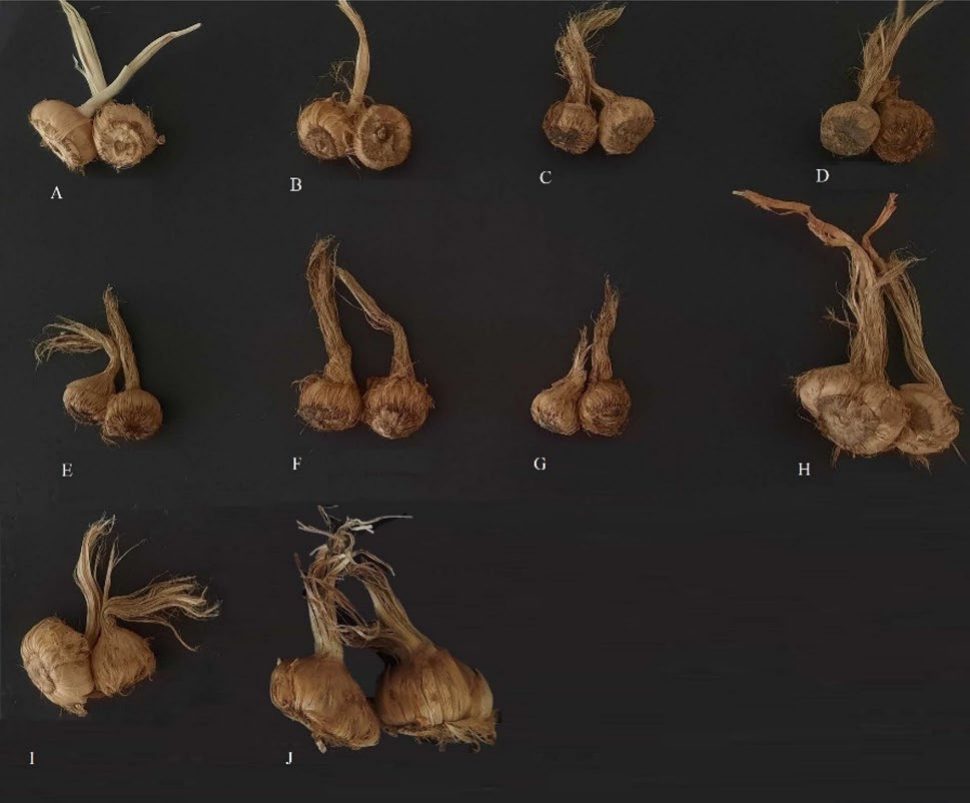
Saffron corms of different ecotypes were used in this study. Isfahan(**A**), Torbat Heydariyeh (**B**), kashmar(**C**), Ferdows(**D**), Shahrekord(**E**), Mashahad(**F**), Ghaen(**G**), Zaveh(**H**), Hamedan(I), Arjenak(J), respectively. The details of geographic characteristic of regions of collected ecotypes were listed in Supplementary Table S4.

The corms of saffron were collected from different geographical locations of traditional saffron production areas in Iran (Fig. 6 and Supplementary Table S4). The taxonomy of the *C. sativus* plant was confirmed by Dr Hamzeh-Ali Shirmardi, from Chaharmahal and Bakhtiari Agricultural and Natural Resources Research and Education Center, Shahrekord, Iran (shirmardi1355@gmail.com). The plant corms and the voucher specimen (Her-barium No.1400402/381) were stored in the collection of medicinal plants of the Department of Agricultural Biotechnology at the Tarbiat Modares University. Different ecotypes of *C. sativus* were grown in a climate-controlled greenhouse under a 10 h photoperiod in Shahr-e Kord, Iran, with coordinates 32.3282° N, 50.8769° E, at the temperature of 14–18 °C and decent loamy soil (20% sand, 30% silt, and 30% clay) with pH 7.8–8.3 from September to December 2021. In order to minimize fungal diseases, triggered chiefly by *Fusarium spp*, 1% fungicide water solution of copper oxychloride was used to treat the corms for 30–60 s. The biological replicates of saffron stigmas were randomly harvested in the early morning hours during the flowering stage (on November 11th, 2021, Supplementary Fig. S8). At this stage, some of the morphological traits such as fresh stigma weight, dried stigma weight, stigma length, corm weight, horizontal diameter, flower fresh weight, and day -to- flower were measured in the studied ecotypes, the result presented in Supplementary Table S3. The fresh stigmas were frozen in liquid nitrogen and preserved at –80 °C for metabolite analysis and RNA extraction.

### High performance liquid chromatography (HPLC) Analysis

The stigma tissues were freeze-dried with a freeze dryer machine (CHRiST model ALPHA 1–4) and then were stored at 4 ◦C until further analysis. Chemical standards of picrocrocin (CAS number 138–55-6, Sigma-Aldrich, St. Louis, MO, USA, ≥ 90.0% purity) and crocins (CAS number 42553–65-1, Sigma-Aldrich, St. Louis, MO, USA) (> 90% purity) were used. 10 mg of stigma tissues were grounded and then homogenized in 10 ml ethanol at 80%. Then samples were sonicated by an ultrasonicator (Stima sonic Company—Iran) for over 15 min in dark conditions at 25 °C. Next, the samples were centrifuged for 5 min at 8000 rpm and filtered following the supernatant was used for chromatography by HPLC/DAD to determine saffron components^88^.

HPLC equipment was performed by KNAUER System equipped with a binary well chrome K1001, a multiple wavelength UV–Vis (DAD)-2800 model KNAUER Eurospher equipped with a 20 μl injection loop and RP C18 (4.6 × 250 mm × 5 µm) the column was used for analytical separation. A combination of acetonitrile (solvent A) and water (solvent B) was applied as a mobile phase with a flow rate of 1.0 ml/min at room temperature. The gradient method was used at 90% for 5 min then decreased to 20% in 20 min and was kept for more than 5 min. The acquisition of the chromatograms was adjusted at different maximum absorption including 308 nm, and 440 nm to determine picrocrocin, and crocins, respectively. The identification of each compound was achieved by spiking its retention time with standards under equal conditions. The quantification analysis was assessed by evaluating the integrated peak area and concentration levels, which was considered using the calibration curve by plotting the peak area against the different concentration levels of the particular standard compounds. Four-point regression curves were obtained in the concentrations of 2.5,10, 50, and 200 ppm for the crocin standard and 25,50, 100, and 200 ppm for the picrocrocin standard. The correlation coefficients (R^2^) were measured for all authentic standards of compounds, which were about 0.99.

#### Validation of selected genes of the modules with qRT-PCR

Based on network analysis results, we selected several identified hub TFs and associated genes from the noticeable apocarotenoid modules for validation by RT-qPCR. 100 mg red stigma tissue in all bulked stigma samples (15 stigmas for each replicate) was used for RNA extraction. Total mRNA extraction was performed using DENAzist Column RNA Isolation Kit (DENAzist Asia Co., S-1010, Mashhad, Iran) and treated with DNase I (Thermo Fisher Scientific, Waltham, MA, USA) to remove remaining genomic DNA contamination according to the manufacturer’s instructions. The RNA quality and integrity were checked using 1.2% agarose gel, and the concentration was examined using Nanodrop (BioTek, EPOCH). Approximately 1 μg of DNase I-treated total RNA was subjected to the cDNA synthesis using Add Scrip cDNA synthesis kit (add-bio, Korea, Lot:22,701) based on the manufacturer’s instructions. Primers were designed by PerlPrimer (version 1.1.21) software^89^ and OligoAnalyzer (Version 3.1) web-based program (https://www.idtdna.com/pages/tools/oligoanalyzer) subsequently, were checked using in silico NCBI Primer-BLAST tool (https://www.ncbi.nlm.nih.gov/tools/primer-blast/) to detect target-specific primers. To prevent non-specific amplification, one of the primers in each pair was designed to 3′ untranslated regions (3′UTRs) region due to the relatively high-level specificity of this region for one specific transcript. The details of primers are in Table 3. Finally, all designed primers were confirmed by standard PCR and 2% agarose gel electrophoresis, verifying primers’ specificity for certain genes within gene families. Tubulin (TUBA) gene was used as the endogenous reference gene according to Qian et al. (2019)^39^. The RT-qPCR reactions were performed on the mini opticon real-time PCR detection system (Bio-Rad). The reaction mixture (10 μl) contained 2 × Power SYBR Green PCR Master mix No ROX (Ampliqon, Inc., Denmark, Lot: A323402-25), 0.75 μl of template cDNA and 2 μM of each primer (forward and reverse). The amplification program was run as follows: 95°C for 15 min, 95°C for 30 s, 58°C for 30 s, 72°C for 30 s and repeated for 40 cycles. The melting curve for products was gained by running at 65–95°C for 5 s after each run. The relative expression levels of target genes were carried out using a relative standard curve based on the threshold of Ct values. Data were evaluated according to the Livak method (2^−ΔΔCt^)^90^, and the graphs were built using the ggplot2 package in RStudio (version 2022.07.2–576). Each technical and biological sample was measured in triplicate.

**Table 3:** The list of primers for hub TFs and related genes used in quantitative Real-Time PCR expression analysis.

Primer name	Sequence (5′ → 3′)	Tm (ºC)	Length	Product length
C.Sa_ADH-11367_F	GGATTTAAGATGAGTGGGCAGG	55.5	22	147
C.Sa_ADH-11367_R	TTCGTCAATGTCTTCTTCGCTC	55.2	22	
C.Sa_MYB_F	CATATTGGGCTTCTCTGGTTCA	54.8	22	145
C.Sa_MYB_R	GGAAGTAAATCACCTCTCCACTG	54.9	23	
C.Sa_ERF_F	TAAGACTGAGGGAGCAAGAGA	54.7	21	182
C.Sa_ERF_R	GCTTCTAGTAGCCAAGGACTT	54.1	21	
C.Sa_UGT73D1_F	TGTCGCTCTACAACAAGGATT	54	21	169
C.Sa_UGT73D2_R	CCTATCTCGATGACCTGCG	54.8	19	
C.Sa_MADS_F	GATATTCTCGGACATGACTCCC	54.5	22	175
C.Sa_MADS_R	AACATATATCGTGGGTTCGACC	54.5	22	
C.Sa_GLT2_F	GAGTTCATTGGTCAGTGTTGC	54.3	21	193
C.Sa_GLT2_R	AATGAAAAGTTAAGCCGTGTGG	54	22	
C.Sa_UGT12_F	ACTTTGCATGGTCACTTCTGG	55.5	21	121
C.Sa_UGT12_R	CCAAATAGCGGAACTCTTGGG	55.9	21	
C.sa_ALDH3F1_F	TGAGTTTAGCTTCAGATACCCAC	54.3	23	143
C.sa_ALDH3F1_R	TATGGATCATTCTATCGGTCTGC	54	23	
C.Sa_bzip_F	CCCATTTCCGCTCATAGAGT	54.3	20	132
C.Sa_bzip_R	GGCATATGTCCAACAACTTGAG	54	22	
C.Sa_C2H2_F	CCGATGGAAGAACTGTGGA	54.4	19	176
C.Sa_C2H2_R	GCAAACCGTGAATTTCGCAA	54.9	20	
C.Sa_CCD2L_F	AGAACCTGTGGCTGTTGTG	55.4	19	130
C.Sa_CCD2L_R	ACATTGTGAGTCCCTAGCAGA	55.5	21	
C.SaTUB_F	GAGAAGGATTACGAAGAGGTGG	54.6	22	152
C.SaTUB_R	TCAACAAAGATAACCGAGGCAT	54.4	22	
C.Sa_HB_F	GGCACGATTGGTTCAGGAAA	55.8	20	156
C.Sa_HB_R	GGGTCATCGTACAAATCCTAGC	55.2	22	

#### Statistical analysis

The RT-qPCR data were analyzed by XLSTAT (XLSTAT 2022) (https://www.xlstat.com/). Partial Least Squares regression (PLS) was performed to determine complex relationships between TFs and dependent genes. The corrplot (version 0.92) package in R was used to visualize a correlation matrix and confidence interval. The confidence level was considered 0.95.

### Supplementary Information

Supplementary Information 1.

Supplementary Information 2.

## Data Availability

The datasets generated during and/or analysed during the current study are available in the National Center for Biotechnology Information (NCBI) repository, https://www.ncbi.nlm.nih.gov/sra. The accession numbers are included: SRR10028150, SRR10028151, SRR5985561, SRR5985560, SRR5985559, SRR5985558, SRR5985557, SRR5985556, SRR5985555, SRR5985554, SRR5985553, SRR1910567, SRR1909704, SRR1909702, SRR1767302, SRR10028145, SRR10028154, SRR8284572, SRR8284574.

## Abbreviations

SM: Secondary metabolite
TF: Transcription factor
WGCNA: Weighted Correlation Network Analysis
IPP: Isopentenyl diphosphate
MVA: Mevalonic acid
MEP: Methyl erythritol 4-phosphate
GGPP: Geranylgeranyl pyrophosphate
PSY: Phytoene synthase
PDS: Phytoene desaturase
Z-ISO: ζ-Carotene isomerase
ZDS: ζ-Carotene desaturase
CRTISO: Carotenoid isomerase
LCYB: Lycopene β-cyclase
BHY: β-Carotene hydrolase
CCD2: Carotenoid cleavage dioxygenase2
ALDH: Aldehyde dehydrogenase
GLTs/UGTs: Glocosyl transferase enzymes
t-SNE: T-distributed stochastic neighbor embedding
TOM: Toplogical overlap matrix
KEGG: Kyoto encyclopedia of genes and genomes
NCBI: National Center for Biotechnology Information
SVA: Surrogate variable analysis
PLS: Partial Least Squares regression
qRT-PCR: Quantitative real-time PCR
HPLC: High performance liquid chromatography
