# Supplementary material for: Co-regulatory network analysis of the main secondary metabolite (SM) biosynthesis in Crocus sativus L
Source: Sci Rep. 2024 Jul 9;14:15839. doi: 10.1038/s41598-024-65870-z (PMC11233700; doi:10.1038/s41598-024-65870-z)
Supplement: Supplementary file 1 — Supplementary Information 1. [file 41598_2024_65870_MOESM1_ESM.docx]

***For submission to Scientific Reports***

**Supplementary Material**

**Co-regulatory network analysis of the main secondary metabolite (SM) bio-synthesis in *Crocus sativus* L.**

Mahsa Eshaghi^1^, Sajad Rashidi-Monfared*^1^

^1^Department of Plant Biotechnology, Faculty of Agriculture, Tarbiat Modares University, Tehran, Iran.

^1^Department of Plant Biotechnology, Faculty of Agriculture, Tarbiat Modares University, Tehran, Iran.

* Corresponding author.

E-mail address: [rashidims@modares.ac.ir](mailto:rashidims@modares.ac.ir) (S. Rashidi-Monfared)

**Table S1.** The details of RNA-seq data resources.

| **SRX Accession** | **SRA Accession** | **tissue** | **method** | **location** | **spot** |
| --- | --- | --- | --- | --- | --- |
| SRX6764381 | SRR10028150 | Stigma | Illumina HiSeq | Italy | 14379856 |
| SRX6764380 | SRR10028151 | Stigma | Illumina HiSeq | Italy | 14,557,929 |
| SRX3141529 | SRR5985561 | Stigma | Illumina HiSeq | China | 21053494 |
| SRX3141528 | SRR5985560 | Stigma | Illumina HiSeq | China | 20321439 |
| SRX3141527 | SRR5985559 | Stigma | Illumina HiSeq | China | 17832058 |
| SRX3141526 | SRR5985558 | Stigma | Illumina HiSeq | China | 15273299 |
| SRX3141525 | SRR5985557 | Stigma | Illumina HiSeq | China | 23044742 |
| SRX3141524 | SRR5985556 | Stigma | Illumina HiSeq | China | 24972464 |
| SRX3141523 | SRR5985555 | Stigma | Illumina HiSeq | China | 16857513 |
| SRX3141522 | SRR5985554 | Stigma | Illumina HiSeq | China | 19659130 |
| SRX3141521 | SRR5985553 | Stigma | Illumina HiSeq | China | 23002038 |
| SRX952266 | SRR1910567 | Stigma | Illumina HiSeq | India | 29521835 |
| SRX951261 | SRR1909704 | Stigma and Flower | Illumina HiSeq | India | 29521835 |
| SRX951262 | SRR1909702 | Stigma and Flower | Illumina HiSeq | India | 37716452 |
| SRX848602 | SRR1767302 | Stigma | Illumina HiSeq | India | 22932750 |
| SRX6764386 | SRR10028145 | Stigma | Illumina HiSeq | Spain | 13124474 |
| SRX6764377 | SRR10028154 | Stigma | Illumina HiSeq | Spain | 18678838 |
| SRX5099343 | SRR8284572 | Stigma | Illumina HiSeq | Spain | 18678838 |
| SRX5099341 | SRR8284574 | Stigma | Illumina HiSeq | Spain | 28470254 |

**Table S2**. list of hub TFs related to apocarotenoids modules.

| **Module** | **Hub TF** |
| --- | --- |
| brown | MADS |
|  | C2H2 |
|  | bZIP |
|  | HD-ZIP |
|  | CO-like (ZFP) |
|  | CO-like |
|  | ERF |
| blue | GATA family |
|  | HD-ZIP |
|  | TALE family (HB) |
| green | bZIP |
|  | Myb |

**Table S3.** Measurement of some morphological traits in the studied ecotypes.

| **Ecotypes\Traits** | **Arjenak** | **Ghaen** | **Mashhad** | **Zaveh** | **Isfahan** | **Hamedan** | **Torbat** | **Kashmar** | **Ferdows** | **ShahreKord** |
| --- | --- | --- | --- | --- | --- | --- | --- | --- | --- | --- |
| Fresh stigma weight (mg) | 46.01±0.29 | 43.47±0.18 | 41.13±0.25 | 38.40±0.13 | 36.04±0.35 | 32.80±0.14 | 30.13±0.88 | 26.85±0.35 | 24.64±0.09 | 21.36±0.33 |
| Dried stigma weight (mg) | 7.49±0.51 | 7.81±0.32 | 7.39±0.045 | 6.90±0.23 | 6.48±0.64 | 5.89±0.26 | 5.42±0.15 | 4.83±0.63 | 4.48±0.17 | 3.93±0.10 |
| Stigma length (mm) | 48.42±0.04 | 43.79±0.01 | 41.46±0.07 | 38.57±0.06 | 37.44±0.07 | 33.49±0.08 | 30.49±0.08 | 29.25±0.07 | 29.08±0.07 | 2.86±0.004 |
| Corm weight (g) | 23.46±1.13 | 9.96±0.18 | 9.09±0.85 | 13.28±0.52 | 10.33±0.41 | 8.03±0.28 | 7.13±0.36 | 5.83±0.21 | 5.34±0.32 | 4.72±0.2 |
| Horizontal diameter (mm) | 39.58±1.61 | 39.06±0.76 | 29.46±1.66 | 35.93±0.71 | 30.70±0.89 | 28.52±1.17 | 23.69±0.16 | 23.63±0.67 | 22.64±0.12 | 21.30±.55 |
| Flower fresh weight (mg) | 575±3.74 | 543±2.28 | 514±3.14 | 480±1.66 | 450±4.42 | 410±1.76 | 0.366±1.84 | 0.335±4.39 | 308±1.22 | 270±3.98 |
| Day to flower (days) | 27 | 48 | 31 | 50 | 51 | 36 | 49 | 46 | 48 | 52 |

**Table S4.** The details of geographic characteristic of regions of the collected saffron ecotypes.

| **Latitude(N)** | **Longitude(E)** | **Elevation(m)** | **Province** | **Region** | **No.** |
| --- | --- | --- | --- | --- | --- |
| 32.65.39° N | 51.6660° E | 1574 | Isfahan | Isfahan | 1 |
| 35.27.98° N | 59.2161° E | 1425 | Razavi-Khorasan | Torbat Heydariyeh | 2 |
| 35.2434° N | 58.4687° E | 1063 | Razavi-Khorasan | Kashmar | 3 |
| 34.0228° N | 58.1722° E | 1293 | South Khorasan | Ferdows | 4 |
| 36.2605° N | 59.6168° E | 995 | Razavi Khorasan | Mashhad | 5 |
| 33.7227° N | 59.1788° E | 1455 | South Khorasan | Ghaen | 6 |
| 350 27° N | 590 47 ° E | 1300 | Razavi Khorasan | Zaveh | 7 |
| 32.3282° N | 50.8769° E | 2,070 | Chahar Mahal Va Bakhtiari | Shahr-ekord | 8 |
| 35.2113° N | 48.7242° E | 1,671 | Hamadan | Kabudrahang | 9 |
| 32.4284° N | 50.6521° E | 2,850 | Chahar Mahal Va Bakhtiari | Arjenak | 10 |


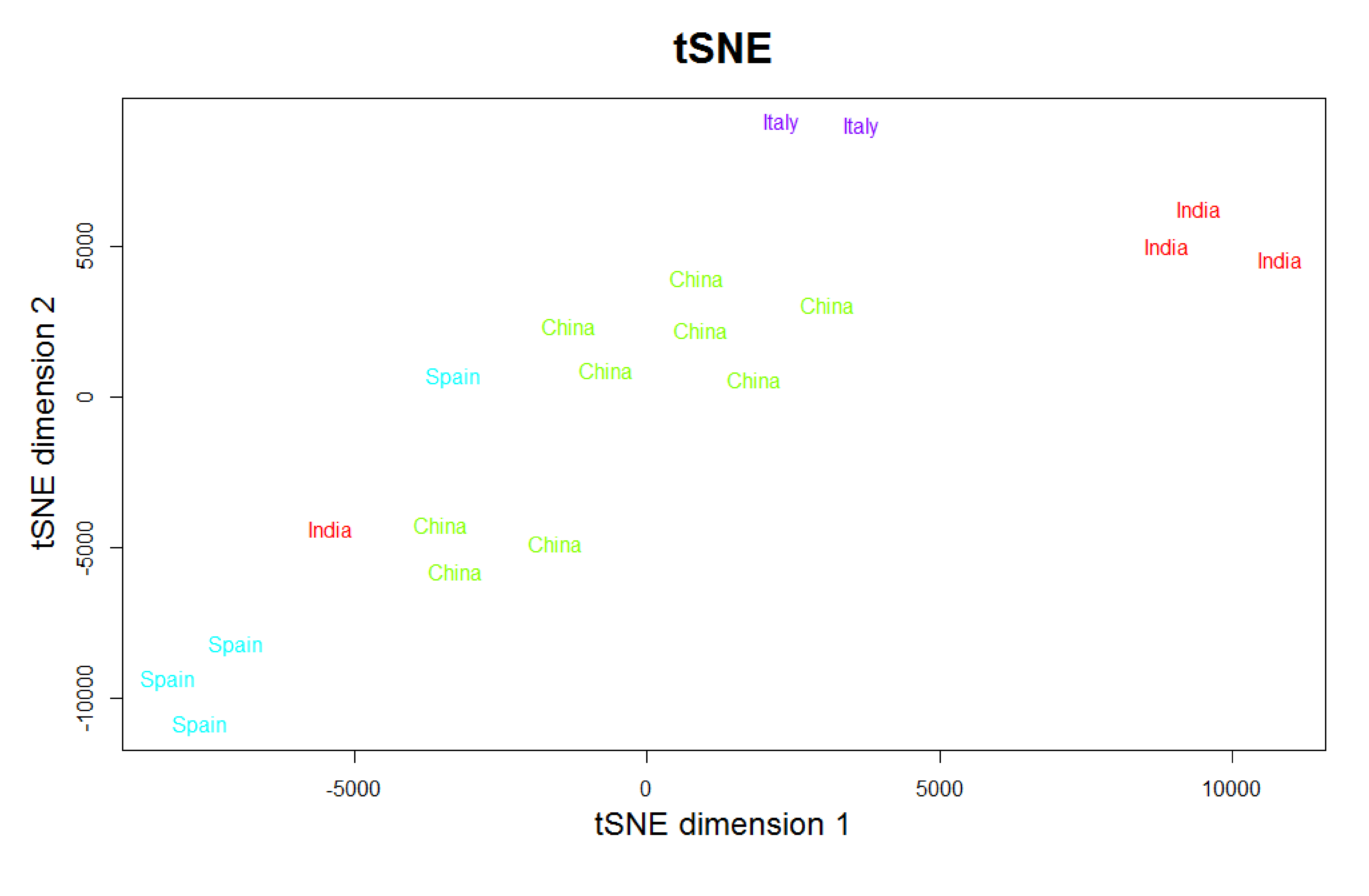


**Figure. S1**. T-SNE analysis to reveal the classification of different ecotypes based on the transcriptome sequencing data.


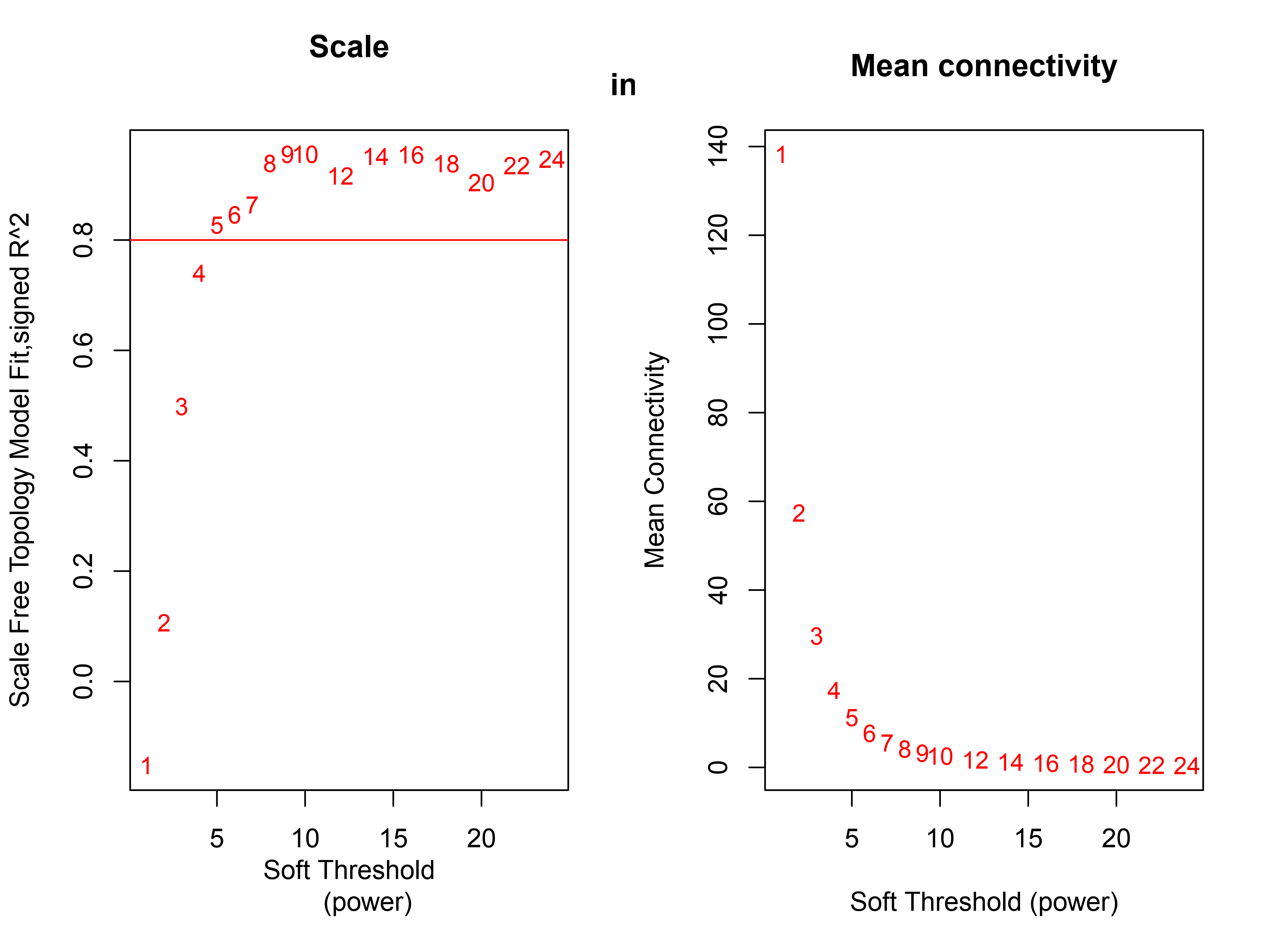


**Figure. S2**. Soft threshold determination method used to gain the scale-free topology index.


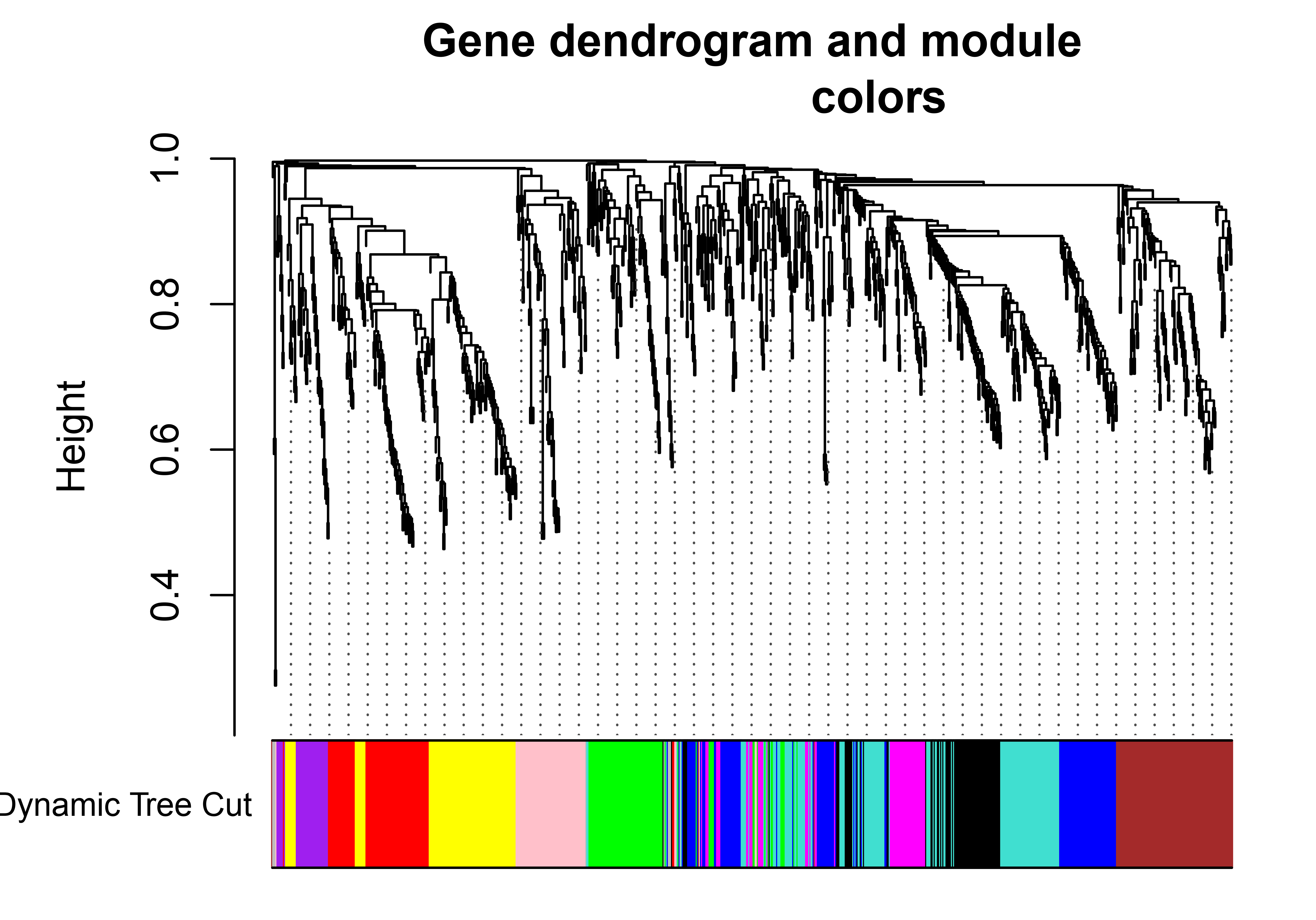


**Figure. S3**. Hierarchical clustering based on dissimilarity measurement is used to detect gene clusters in order to discover modules in WGCNA. The colored row below the dendrogram shows, modules were determined, as branches of a cluster tree, and each module was considered by a specific color using the dynamic tree cut method.


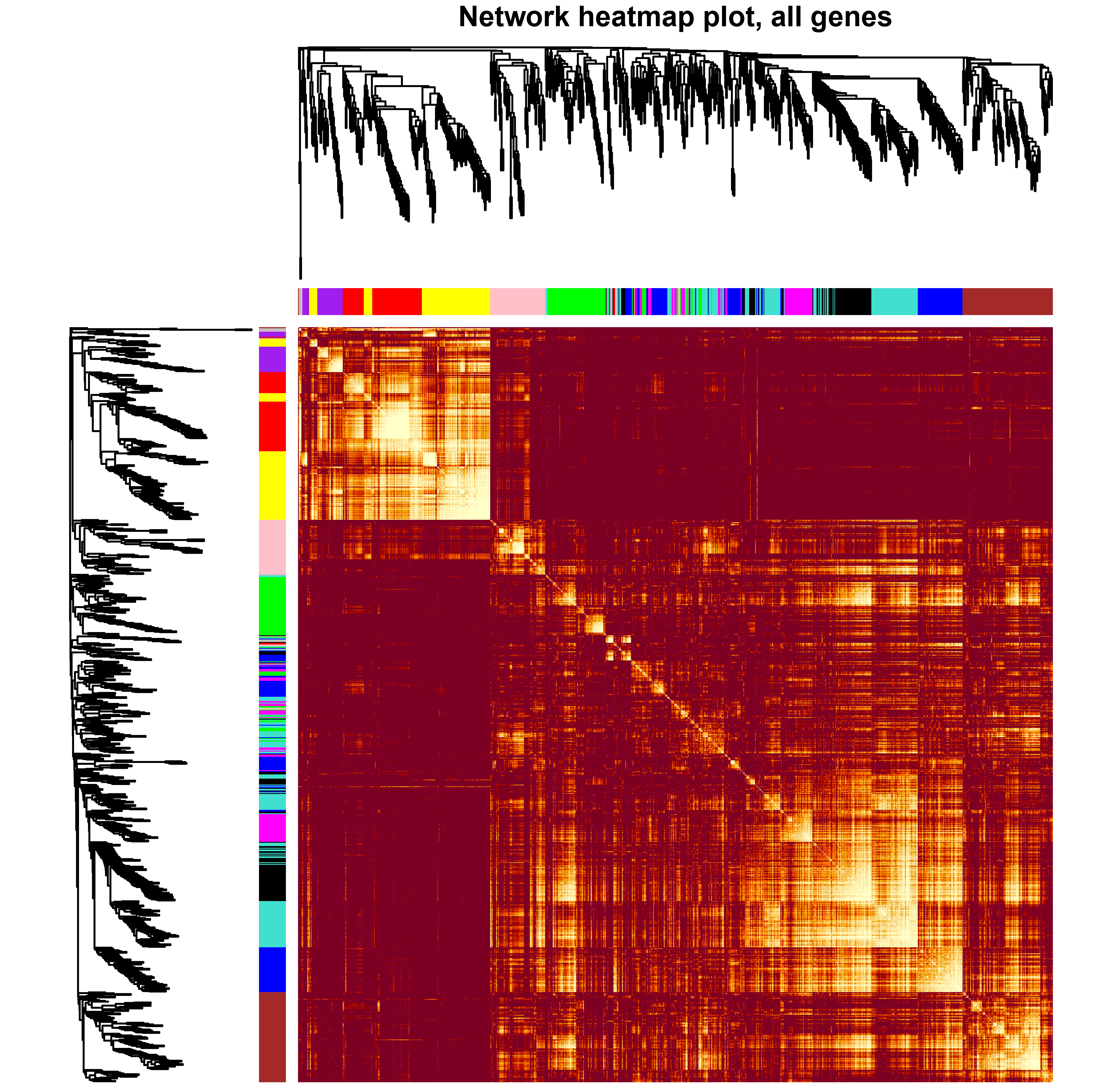


**Figure. S4.** Side view of topological overlap heatmap plot in the gene network. Light and dark colors represent low and high topological overlap, respectively. The module assignment and gene dendrogram are also exposed the top and left side.

**Figure. S5.** Protein-protein interactions network of hub TFs related to apocarotenoids modules including blue module (A), brown module (B), and green module (C) in *C. sativus* based on string analysis are shown, respectively.


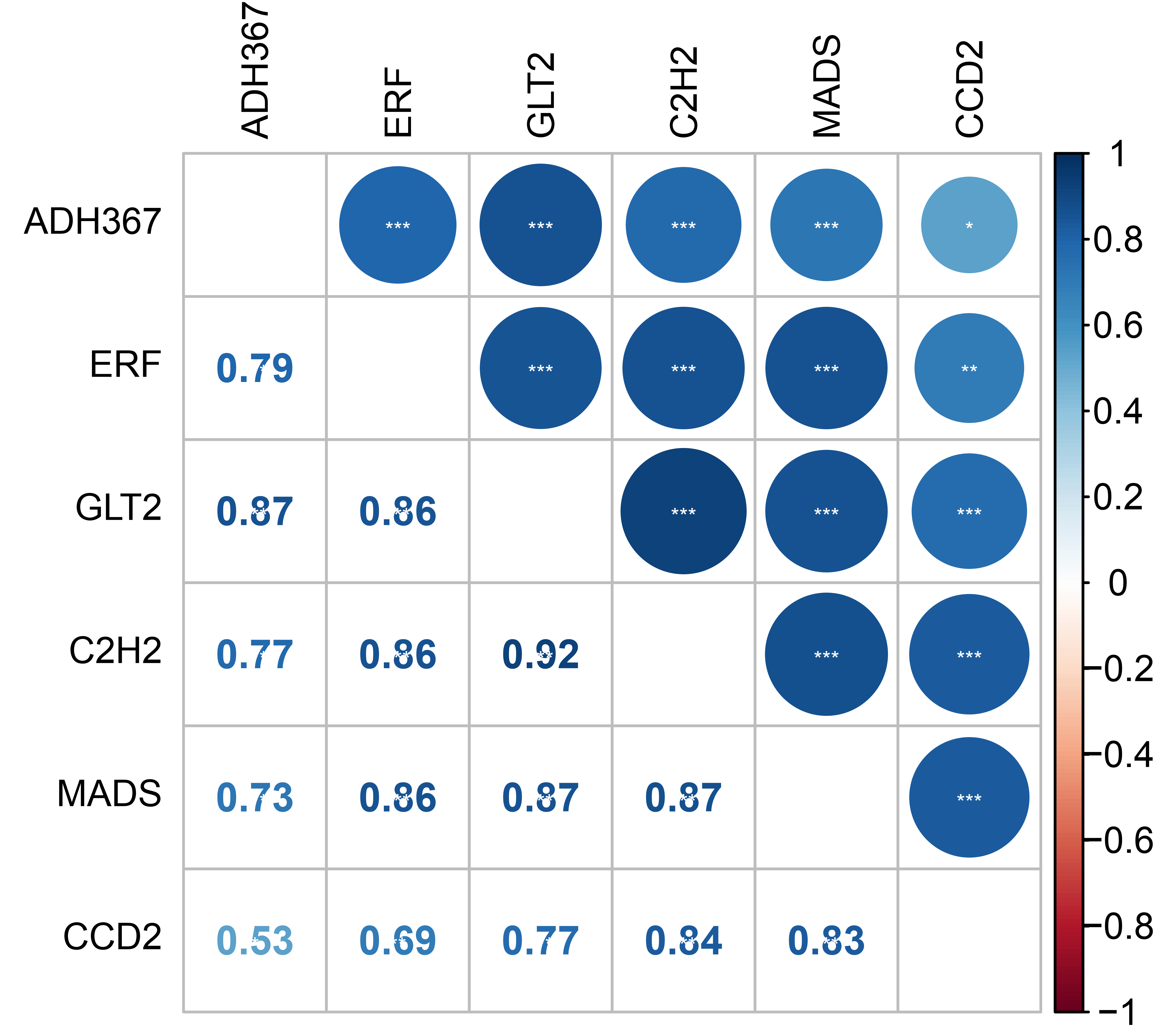


**Figure. S6.** A graphical representation of a correlation matrix, confidence interval, and significant levels between hub TFs and genes in brown module.


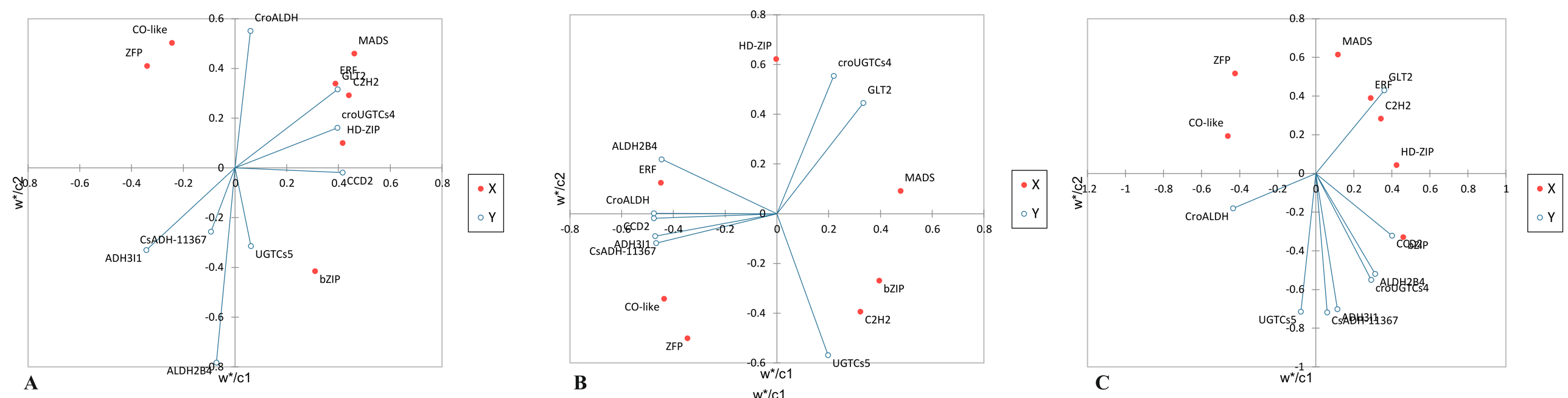


**Figure. S7.** Partial Least Squares regression (PLS) was carried out for determining complex relationships between TFs and dependent genes. The results showed the strong effect of hub TFs such as MADS, C2H2, ERF, and HD-ZIP on related apocarotenoid genes especially CCD2 and GLT2. PLS analysis in total (RED, –2 DAY, and 0 DAY)- (A), high (–2 DAY, and 0 DAY)- (B), low (RED stage)- (C) content metabolite and stigma developmental stages.


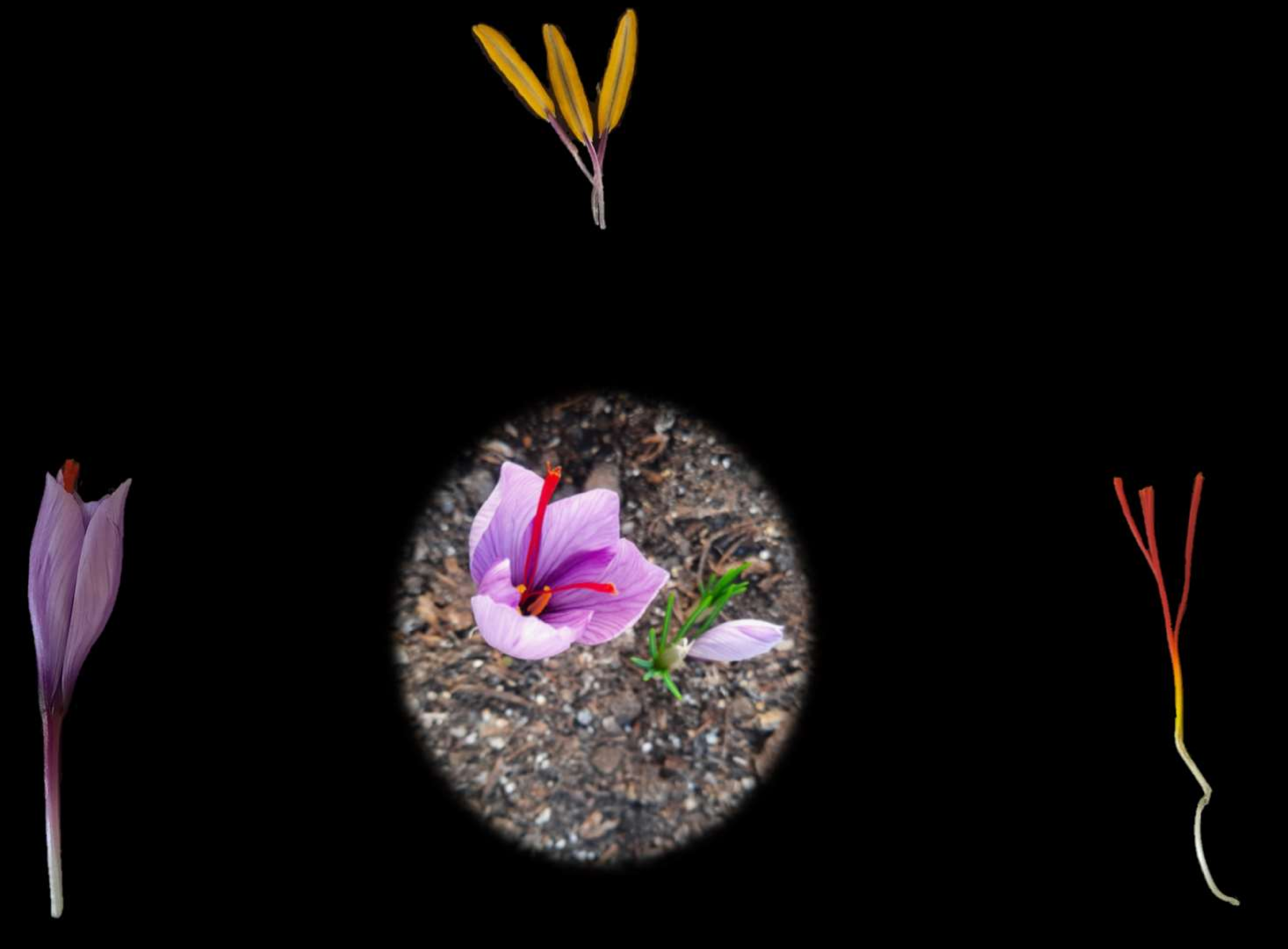


**Figure. S8.** Sampling red saffron stigmas during the flowering stage.
